# Mapping aplastic anaemia hospital activity in England

**DOI:** 10.1002/jha2.869

**Published:** 2024-03-22

**Authors:** Bamidele Famokunwa, Aman Gupta, Stephen Thomas, Morag Griffin, Austin Kulasekararaj

**Affiliations:** ^1^ Pfizer Limited Tadworth Surrey UK; ^2^ Wilmington Healthcare London UK; ^3^ St. James's Hospital, Leeds Teaching Hospitals NHS Trust Leeds UK; ^4^ King's College Hospital NHS Foundation Trust London UK; ^5^ King's College London London UK

**Keywords:** aplastic anaemia, bone marrow failure, epidemiology, paediatric aplastic anaemia, stem cell transplantation

1

Aplastic anaemia (AA) is a rare, life‐threatening, haematological disease [1, 2]. In almost all cases, the cause is idiopathic [1]. AA has an incidence of 2–3/million, with a bimodal peak (teenagers and older adults), and a similar prevalence in males and females [2]. Incidence is up to five times higher in Asia than in Europe [1, 3, 4]. Treatment includes haematopoietic stem cell transplant and immunosuppressive therapy [5, 6].

There is a need to evaluate the specific UK incidence data on AA. In this study, instead of a registry, we used hospital admission data from the Hospital Episode Statistics (HES) database* to understand the population of newly hospitalised AA patients in England. We identified people admitted to the hospital between 1 April 2017 and 31 March 2022 with an International Classification of Diseases version 10 (ICD‐10) code of D61 (‘Other AAs’). See the Supporting Information Materials for methodology.

The most frequent diagnosis was ‘AA, unspecified’ (D619) (Table 1). A diagnosis of ‘constitutional AA’ (D610) was given to 12–13% of the cohort each year. The number of admissions for constitutional AA in 2017/2018 was higher than in subsequent years of the survey (60 versus 10–15), likely explained by the use of a defined five‐year window to collect data on the first instance of a diagnosis. In years 2–5 (i.e., 18/19‐21/22) of the study we had access to historical data to determine that any presentation was indeed a first presentation. Over these 4 years (18/19‐21/22), 715 new AA diagnoses were identified, Year 1 (17/18) was excluded due to the likely overestimate of the total patients admitted to the hospital in that year. There was an average of approximately 180 new cases per year over years 2–5, based on rounded annual values.

**TABLE 1:** New patients admitted to hospital each year with a diagnosis of aplastic anaemia 2017/2018–2021/2022^†^ **^.^**

Aplastic anaemia codes		New patients* by fiscal year						
ICD‐10 code	Diagnosis description	2017/2018	2018/2019	2019/2020	2020/2021	2021/2022	Annual average (year 2–5)**	5 years total
D610	Constitutional AA	60	10	15	10	15	15	115
D611	Drug‐induced AA	*	*	*	*	*	*	15
D612	AA due to other external agents	0	0	0	*	*	0	*
D613	Idiopathic AA	*	0	*	*	0	*	*
D618	Other specified AAs	*	*	*	*	10	*	20
D619	AA, unspecified	160	150	145	155	170	155	785
D61	Other AAs	225	170	165	175	205	180	940

*Notes*: All counts were rounded to the nearest 5. *Suppressed data. **Annual average based on rounded annual values for years 2–5 with year 1 excluded due to likely overestimate of total patients.

^†^ This study uses data provided by patients and collected by the NHS as part of their care and support. Secondary care data is taken from the English Hospital Episode Statistics (HES) database produced by NHS England, Copyright © 2024, NHS England. Re‐used with the permission of NHS England. All rights reserved. See the Supporting Information Materials for a full disclaimer.

The highest incidences of AA were in central southern England and the north of England (Figure S1).

Patients’ ages at first hospitalisation ranged from 0 to 100, with the majority aged over 40 (Figure 1). In the constitutional AA group, the majority were aged under 40. The proportions of males and females in 5‐year age bands up to age 50 were similar.

**FIGURE 1:**
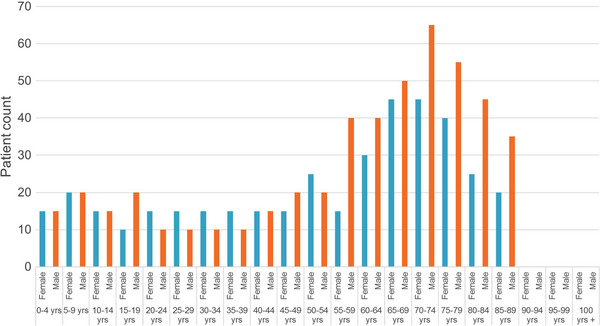
First hospitalisation for patients with aplastic anaemia by 5‐year age bands and gender (2017/2018–2021/2022)**^†.^** ^†^This study uses data provided by patients and collected by the NHS as part of their care and support. Secondary care data is taken from the English Hospital Episode Statistics (HES) database produced by NHS England, Copyright © 2024, NHS England. Re‐used with the permission of NHS England. All rights reserved. See the Supporting Information Materials for a full disclaimer. Patient counts between 1 and 7 (inclusive) have been suppressed.

Overall, 74% of patients were white. The ethnicity with the highest incidence rate was ‘Any other Asian background’ (i.e. those without Indian, Pakistani, Bangladeshi, or Chinese background) at 27 patients per 1,000,000 population (see Table S1].

Sixty‐five patients (7%) underwent a haematopoietic stem cell transplant – an average of 15 per year. The mean time from diagnosis to transplant was 163 days. Ninety‐two percent of allogeneic transplants had peripheral blood as stem cell source, whilst the remainder were bone marrow‐derived stem cell transplants. Among those who received transplants, 69% of recipients were aged under 40. Most patients aged under 40 (81%) did not receive a transplant. 3% of patients aged over 40 received a transplant. The frequency of transplants did not vary across ethnic groups.

Compared with previous studies in patients with AA, this study included a relatively large number of patients [2–4, 7–9]. Its findings, including incidence rate and age distribution for constitutional AA, align with previously published research [7, 8]. As in other reports, the majority of patients were aged over 60. There was a suggestion of a higher predominance in the under‐20s, but this was not as high as previously reported.

Our finding of higher proportions of males in each 5‐year age band above 50 years is supported by some previous studies [3, 9], though some studies have shown a predominance of females [7, 8, 10]. Studies in Asia have found a higher incidence of AA than in the West, which is understood to be due to environmental factors [3, 4]. The high incidence of AA in the ‘Any other Asian background’ in our study needs further analysis to evaluate whether this is driven by exposure to different environmental factors in patients who have lived outside England.

Although the number of patients undergoing transplantation was small, there was no evidence of differences in incidence across racial groups. Treatment includes haematopoietic stem cell transplant and immunosuppressive therapy based on age and donor compatibility [1, 4, 11]. Studies from Asia and Europe have found that ≤10% of patients of all ages receive first‐line HSCT, which is supported by our finding of 7% [7, 11–12]. In China, it was suggested this was due to a lack of sibling donors and high costs [11]. Future UK research should assess donor–recipient profiles and the number of patients unable to undergo haematopoietic transplantation due to the lack of a suitable donor. The types and frequencies of other procedures during the study period are consistent with standard clinical practice in patients presenting with symptoms characteristic of AA [7].

This study has some limitations. HES, an administrative dataset, comprises data from hospital trusts across England only. There are variations in data classification and collection across the country, meaning data entry is not geographically uniform. The quality of the coding and the clinical interpretation of the code may vary. The dataset only contains diagnosis‐level data for hospital admissions, meaning that patients treated entirely as an outpatient or in the community are excluded. The nature of AA diagnosis and care in the UK means it is unlikely that many new patient diagnoses or major interventions were missed, as these usually occur during a hospital admission. Prescribing data in secondary care in England is not readily available within the dataset so there is no analysis of patients receiving IST. It also had no comprehensive information on patient outcomes, deaths, and long‐term survival. Suppression was applied to the analysis to prevent the identification of individuals with this rare condition and patient counts with values greater than seven were rounded to the nearest five.

In conclusion, although there are limitations, this paper provides the first epidemiology data mapping patients with AA and their hospital activity in England. To our knowledge, there is no dataset in the UK that aligns the severity of AA with treatment and outcomes. A national registry would allow this level of data to be captured, facilitating more comprehensive surveillance of AA.

## AUTHOR CONTRIBUTIONS

Bamidele Famokunwa, Morag Griffin, Aman Gupta, and Austin Kulasekararaj designed the study, interpreted the data, and drafted and critically revised the manuscript. Stephen Thomas analysed the data and drafted and critically revised the manuscript.

## CONFLICT OF INTEREST STATEMENT

B.F. and A.G. are employees of Pfizer Limited. S.T. is a data analyst employed by Wilmington Healthcare whose work on this project was funded by Pfizer Limited. M.G. has received speaker fees/honoraria from Novartis, Alexion, AstraZeneca, and Sobi. She has contributed to advisory boards for Amgen, Novartis, Sobi, Alexion, AstraZeneca, and Biocryst and provides consultancy to Regeneron and Biocryst. A.K. has received research support for his institution from Celgene/BMS and Novartis. He has acted as a consultant for Samsung, Novo Nordisk, Alexion/AstraZeneca, Arrowhead, and Silence Therapeutics. He has received speaker fees from Alexion/AstraZeneca, Amgen, Celgene/BMS, Pfizer, Novartis, Ra Pharma/UCB, Roche, SOBI, and Janssen. He has contributed to scientific advisory boards and data monitoring committees for Alexion/Astra Zeneca, Apellis, Amgen, Agios, Biocryst, Celgene/BMS, Geron, Novartis, Pfizer, Regeneron, Roche, SOBI, and Janssen.

## FUNDING INFORMATION

The gathering and analysis of data for this analysis was funded by Pfizer Limited. Medical writing assistance was provided by Jane Tricker on behalf of Wilmington Healthcare and funded by Pfizer Limited.

## ETHICS APPROVAL

The authors have confirmed ethical approval statement is not needed for this submission.

## CLINICAL TRIAL REGISTRATION

The authors have confirmed clinical trial registration is not needed for this submission.

## PATIENT CONSENT STATEMENT

The authors have confirmed patient consent statement is not needed for this submission.

## Supporting information

Supporting Information

Supporting Information

Supporting Information

## Data Availability

Not applicable.

## Footnotes

* This study uses data provided by patients and collected by the NHS as part of their care and support. Secondary care data are taken from the English Hospital Episode Statistics (HES) database produced by NHS England, Copyright © 2024, NHS England. Re‐used with the permission of NHS England. All rights reserved. See the Supporting Information Materials for a full disclaimer.
