# Supplementary material for: Mapping aplastic anaemia hospital activity in England
Source: EJHaem. 2024 Mar 22;5(2):414–7. doi: 10.1002/jha2.869 (PMC11020100; doi:10.1002/jha2.869)
Supplement: Supplementary file 1 — Supporting Information [file JHA2-5-414-s003.docx]

**SUPPLEMENTARY MATERIALS**

# Methods

In the absence of a national, disease-specific registry for AA in the UK, the primary source for this analysis was the Hospital Episode Statistics (HES) database for the period 1st April 2017–31st March 2022. Access to licenced HES data was provided through Wilmington Healthcare; see Section 2 for a full disclaimer relating to the use of HES data.

Additional information was derived from the Office of National Statistics (ONS) Population Estimates by Ethnic Group and Religion for England and Wales, 2019 (Experimental Statistics); English Indices of Deprivation, 2019; and the Quality and Outcomes Framework (QOF). The HES database contains details about hospital admissions (including the duration of any continuous stay in the same hospital – defined as a ‘spell’), A&E attendances and outpatient appointments at NHS hospitals and Integrated Care Systems (ICS) in England. Each HES record includes clinical information, demographic information, administrative information, and geographical information for the individual patient.

The HES database was interrogated to find people admitted to hospital with an ICD-10 D61 code (other aplastic anaemias) during the period covered by the analysis. The D61 code includes: constitutional aplastic anaemia (D610), drug-induced aplastic anaemia (D611), aplastic anaemia due to other external agents (D612), idiopathic aplastic anaemia (D613), other specified aplastic anaemias (D618), and aplastic anaemia, unspecified (D619).

In a bid to better define the AA patient cohort within the dataset isolated using the ICD-10 D61 code, patients were also required to have a bone marrow biopsy (Operating Procedure Codes Supplement [OPCS] code W365) and a blood transfusion (OPCS X331, X333, X338, X339) during the period of interest. Patients were excluded if they had a diagnosis of malignancy (multiple myeloma and malignant plasma cell neoplasms [ICD-10 C90], lymphoid leukaemia [ICD-10 C91], myeloid leukaemia [ICD-10 C92], monocytic leukaemia [ICD-10 C93], other leukaemias of specified cell type [ICD-10 C94]), myelodysplastic syndromes (ICD-10 D46), sepsis unspecified (ICD-10 A419) or thrombocytopenia unspecified (ICD-10 D696).

The resulting cohort was stratified by demographics (age, gender, and broad ethnic group), treatment history and geographic location. In this analysis, the term ‘procedure’ in a patient’s treatment history included patient assessments and administration of medicines occurring within 6 months (≤180 days) of their initial D61 hospital admission.

### Rounding and suppression

Patient counts, spell counts and procedure counts with values greater than seven are rounded to the nearest five: this means totals may not sum across columns/rows.

Suppression is a disclosure limitation method: it involves the removal of data from a cell or a row in a table to prevent the identification of individuals in small groups or individuals with unique characteristics. In this analysis, patient counts and procedure counts with values between one and seven (inclusive) are suppressed. Procedure counts per patient are suppressed whenever patient counts are suppressed.

The data available on AA hospitalisation in England is limited and this study was of a descriptive nature only.

# HES disclaimer

1. Secondary care data is taken from the English Hospital Episode Statistics (HES) database produced by NHS Digital, the new trading name for the Health and Social Care Information Centre (HSCIC) Copyright © 2024, the Health and Social Care Information Centre. Re-used with the permission of the Health and Social Care Information Centre. All rights reserved.

 2. HES Data must be used within the licencing restrictions set by NHS Digital, which are summarised below. Wilmington Healthcare accept no responsibility for the inappropriate use of HES data by your organisation.

2.1. One of the basic principles for the release and use of HES data is to protect the privacy and confidentiality of individuals. All users of HES data must consider the risk of identifying individuals in their analyses prior to publication/release.

 2.1.1. Data should always be released at a high enough level of aggregation to prevent others being able to ‘recognise' a particular individual. To protect the privacy and confidentiality of individuals, Wilmington Healthcare have applied suppression to the HES data - ‘*’ or ‘-1’ represents a figure between 1 and 7. All other potentially identifiable figures (e.g. patient numbers, spell counts) have been rounded to the nearest 5.

 2.1.2. On no account should an attempt be made to decipher the process of creating anonymised data items.

 2.2. You should be on the alert for any rare and unintentional breach of confidence, such as responding to a query relating to a news item that may add more information to that already in the public domain. If you recognise an individual while carrying out any analysis you must exercise professionalism and respect their confidentiality.

 2.3 If you believe this identification could easily be made by others you should alert a member of the Wilmington Healthcare team using the contact details below. While appropriate handling of an accidental recognition is acceptable, the consequences of deliberately breaching confidentiality could be severe.

 2.4. HES data must only be used exclusively for the provision of outputs to assist health and social care organisations.

 2.5. HES data must not be used principally for commercial activities. The same aggregated HES data outputs must be made available, if requested, to all health and social care organisations, irrespective of their value to the company.

 2.6. HES data must not be used for, including (but not limited to), the following activities:

 2.6.1. Relating HES data outputs to the use of commercially available products. An example being the prescribing of pharmaceutical products

 2.6.2. Any analysis of the impact of commercially available products. An example being pharmaceutical products

2.6.3. Targeting and marketing activity

 2.7. HES data must be accessed, processed and used within England or Wales only. HES data outputs must not be shared outside of England or Wales without the prior written consent of Wilmington Healthcare.

 2.8. If HES data are subject to a request under the Freedom of Information Act, then Wilmington Healthcare and NHS Digital must be consulted and must approve any response before a response is provided.

 3. 2022/23 HES data are provisional and may be incomplete or contain errors for which no adjustments have yet been made. Counts produced from provisional data are likely to be lower than those generated for the same period in the final dataset. This shortfall will be most pronounced in the final month of the latest period, e.g. September from the April to September extract. It is also probable that clinical data are not complete, which may in particular affect the last two months of any given period. There may also be errors due to coding inconsistencies that have not yet been investigated and corrected.

 4. ICD-10 codes, terms and text © World Health Organization, 1992-2024

5. The OPCS Classification of Interventions and Procedures, codes, terms and text is Crown copyright (2024) published by NHS Digital, the new trading name for the Health and Social Care Information Centre, and licensed under the Open Government Licence.

6. English Indices of Deprivation 2019 data are published by MHCLG (https://www.gov.uk/government/statistics/english-indices-of-deprivation-2019) and licensed under the Open Government Licence.

7. Contains public sector information licensed under the Open Government Licence v3.0. A copy of the Open Government Licence is available at www.nationalarchives.gov.uk/doc/open-government-licence/open-government-licence.htm

8. No part of this database, report or output shall be reproduced or distributed in any form or by any means, or stored in a database or retrieval system, without the prior written permission of Wilmington Healthcare Ltd. Information in this database is subject to change without notice. Access to this database is licensed subject to the condition that it shall not, by way of trade or otherwise, be lent, resold, hired out, or otherwise circulated in any form without prior consent of Wilmington Healthcare Ltd.

9. Whilst every effort has been made to ensure the accuracy of this database, Wilmington Healthcare Ltd makes no representations or warranties of any kind, express or implied, about the completeness, accuracy, reliability or suitability of the data. Any reliance you place on the data is therefore strictly at your own risk. Other company names, products, marks and logos mentioned in this document may be the trademark of their respective owners.

10. You can contact Wilmington Healthcare by telephoning 0845 121 3686, by e-mailing client.services@wilmingtonhealthcare.com or by visiting www.wilmingtonhealthcare.com

# Supplementary tables

**Supplementary Table 1. Patients admitted to hospital with a diagnosis of aplastic anaemia by ethnicity, 5-year total (2017/2018 to 2021/2022)**

| **Ethnicity** | **Patient Count** | **Total Population** | **Patients per 1,000,000 Population** |
| --- | --- | --- | --- |
| British (White) | 665 | 43,660,800 | 15 |
| Not stated | 80 | NA | NA |
| Any other White background | 30 | 3,414,600 | 9 |
| Not known | 35 | NA | NA |
| Indian (Asian or Asian British) | 25 | 1,647,000 | 15 |
| Any other ethnic group | 20 | 1,086,600 | 18 |
| Pakistani (Asian or Asian British) | 20 | 1,327,900 | 15 |
| Any other Asian background | 20 | 745,500 | 27 |
| African (Black or Black British) | 15 | 1,335,600 | 11 |
| Caribbean (Black or Black British) | * | 601,800 | * |
| Irish (White) | * | 342,100 | * |
| Any other Black background | 0 | 128,700 | 0 |
| Bangladeshi (Asian or Asian British) | 10 | 605,000 | 17 |
| Chinese (other ethnic group) | * | 335,600 | * |
| Any other Mixed background | * | 304,800 | * |
| White and Asian (Mixed) | * | 294,300 | * |
| White and Black Caribbean (Mixed) | * | 300,700 | * |
| White and Black African (Mixed) | * | 156,000 | * |

All counts were rounded to the nearest 5. *Suppressed data.

This work uses data provided by patients and collected by the NHS as part of their care and support. Secondary care data is taken from the English Hospital Episode Statistics (HES) database produced by NHS England, Copyright © 2024, NHS England. Re-used with the permission of NHS England. All rights reserved. See Section 2 of the Supplementary Materials for a full disclaimer.

**Supplementary Table 2. Top 20 most common procedures performed at initial ICD-10 D61 hospital admission, 5-year total (2017/2018 to 2021/2022)**

| **Rank** | **Procedure** | **Number of patients, n** | **Proportion of all patients with AA, %** | **Number of procedures, n** |
| --- | --- | --- | --- | --- |
| **1** | Diagnostic extraction of bone marrow* | 345 | 37 | 460 |
| **2** | Computed tomography* | 150 | 16 | 250 |
| **3** | Radiology with post contrast | 140 | 15 | 240 |
| **4** | Pelvis* | 140 | 15 | 225 |
| **5** | Intravenous blood transfusion of packed cells | 115 | 12 | 120 |
| **6** | Radiology of one body area (or for <20 minutes) | 105 | 11 | 230 |
| **7** | Radiology of three body areas (or 20-40 minutes) | 70 | 7 | 110 |
| **8** | Transthoracic echocardiography | 60 | 6 | 95 |
| **9** | Other blood transfusion, unspecified | 60 | 6 | 65 |
| **10** | Radiology of four body areas | 45 | 5 | 70 |
| **11** | Approach to organ under ultrasonic control | 40 | 4 | 60 |
| **12** | Computed tomography of head | 40 | 4 | 75 |
| **13** | Intravenous blood transfusion of platelets | 40 | 4 | 40 |
| **14** | Radiology of two body areas | 40 | 4 | 70 |
| **15** | Blood withdrawal, unspecified | 30 | 3 | 35 |
| **16** | Percutaneous transluminal peripheral insertion of central catheter | 25 | 3 | 35 |
| **17** | Continuous intravenous infusion of therapeutic substance* | 20 | 2 | 20 |
| **18** | Assessment by uni-professional team* | 15 | 2 | 20 |
| **19** | Magnetic resonance imaging* | 15 | 2 | 20 |
| **20** | Fibreoptic endoscopic examination of upper gastrointestinal | 15 | 2 | 30 |

*NEC, not elsewhere classified.

This work uses data provided by patients and collected by the NHS as part of their care and support. Secondary care data is taken from the English Hospital Episode Statistics (HES) database produced by NHS England, Copyright © 2024, NHS England. Re-used with the permission of NHS England. All rights reserved. See Section 2 of the Supplementary Materials for a full disclaimer.

# Supplementary figure

**Supplementary Figure 1. Patients admitted to hospital in England with a diagnosis of aplastic anaemia by Integrated Care Systems (ICS), 5-year total (2017/2018 to 2021/2022): A) number per ICS, B) patients per 100,000 population**

Data for Somerset ICS is suppressed as the total patient count over 5 years is less than or equal to 7.

This work uses data provided by patients and collected by the NHS as part of their care and support. Secondary care data is taken from the English Hospital Episode Statistics (HES) database produced by NHS England, Copyright © 2024, NHS England. Re-used with the permission of NHS England. All rights reserved. See Section 2 of the Supplementary Materials for a full disclaimer.
