# Supplementary figures and images for: Mapping aplastic anaemia hospital activity in England
Source: EJHaem. 2024 Mar 22;5(2):414–7. doi: 10.1002/jha2.869 (PMC11020100; doi:10.1002/jha2.869)

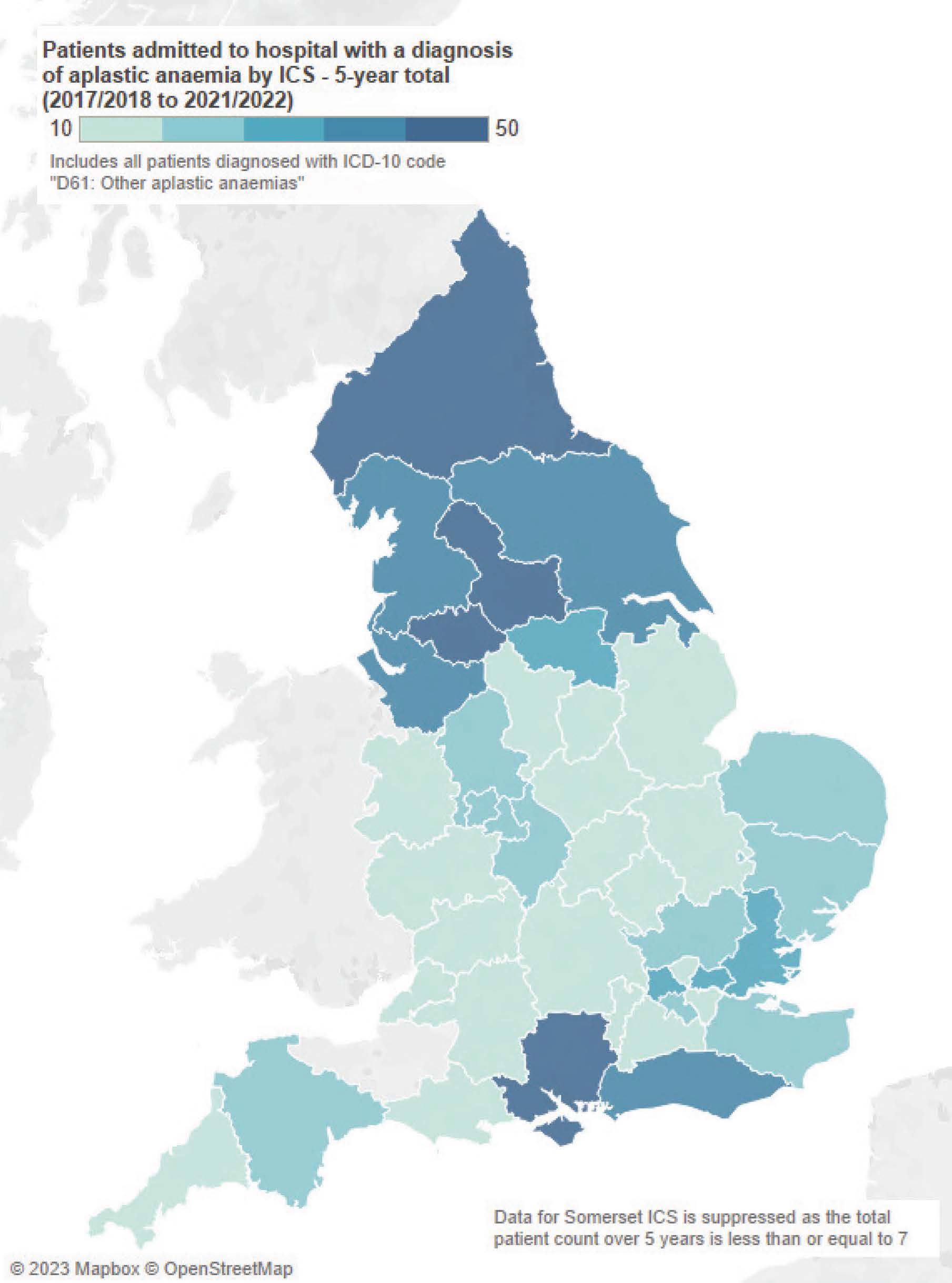

Supplement: Supplementary file 2 — Supporting Information [file JHA2-5-414-s002.jpg]

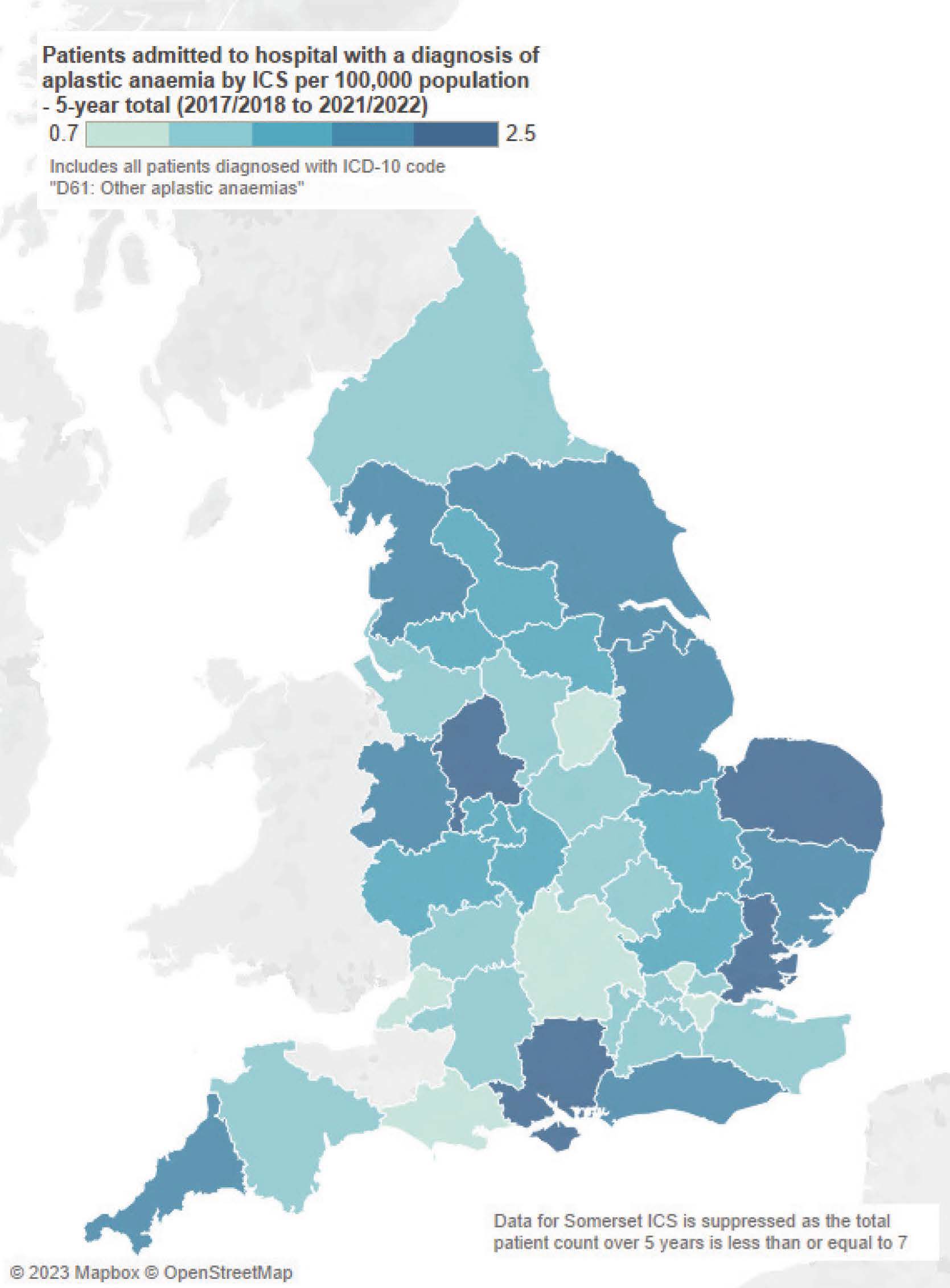

Supplement: Supplementary file 3 — Supporting Information [file JHA2-5-414-s001.jpg]
